# Iatrogenic Injury to Profunda Femoris During a Dynamic Hip Screw Fixation for Intertrochanteric Fracture

**DOI:** 10.7759/cureus.33894

**Published:** 2023-01-17

**Authors:** Anuj Lal, Edwin Jesudason, Rhodri Gwyn

**Affiliations:** 1 Trauma and Orthopaedics, Ysbyty Gwynedd, Bangor, GBR; 2 Trauma and Orthopaedics, University Hospitals Sussex NHS Foundation Trust, Sussex, GBR

**Keywords:** iatrogenic complication, lower extremity vascular injury, dynamic hip screw (dhs), intertrochanteric hip fracture, profunda femoris

## Abstract

We present a case of a profunda femoris artery injury during a dynamic hip screw fixation for an intertrochanteric fracture. This was identified clinically on the ward and confirmed with a CT angiogram. The bleeder was then treated by coil embolization, and laboratory results showed significant improvement in hemoglobin level after blood transfusion.

## Introduction

Injury to the profunda femoris artery (PFA) has been attributed mostly to iatrogenic causes, mostly due to the use of drill bits and sharp bone ends that lead to the development of pseudoaneurysm and haemorrhage from the vessel [1]. Pseudoaneurysm of the vessel can be caused by blunt injury in an atherosclerotic vessel or penetrating trauma to the vessel which can be caused by a bone spike or during surgical intervention [2]. Keel et al. described an injury to the PFA following surgical intervention in intertrochanteric fractures [3].

The last two decades have seen a shift in the management of these injuries from surgical intervention to endovascular techniques. In his paper, Sadat et al. concluded that this drift towards endovascular techniques was mainly due to lesser hospital stay after an endovascular procedure compared to a surgical procedure and because of difficult surgical access to the profunda femoris deep within the muscles [4]. On the contrary Tiwari et al. concluded that surgical treatment in form of an aneurysmectomy and arterial repair should be used to treat pseudoaneurysms of the femoral artery following femur fracture [5].^ ^The incidence of haemorrhage from PFA following dynamic hip screw (DHS) fixation for intertrochanteric fracture has been reported as 0.2% of all PFA injuries by Lazarides et al. [6].

Here, we are reporting a case of iatrogenic injury to the PFA during DHS fixation of extracapsular proximal femur fracture.

## Case presentation

**Figure 1:**
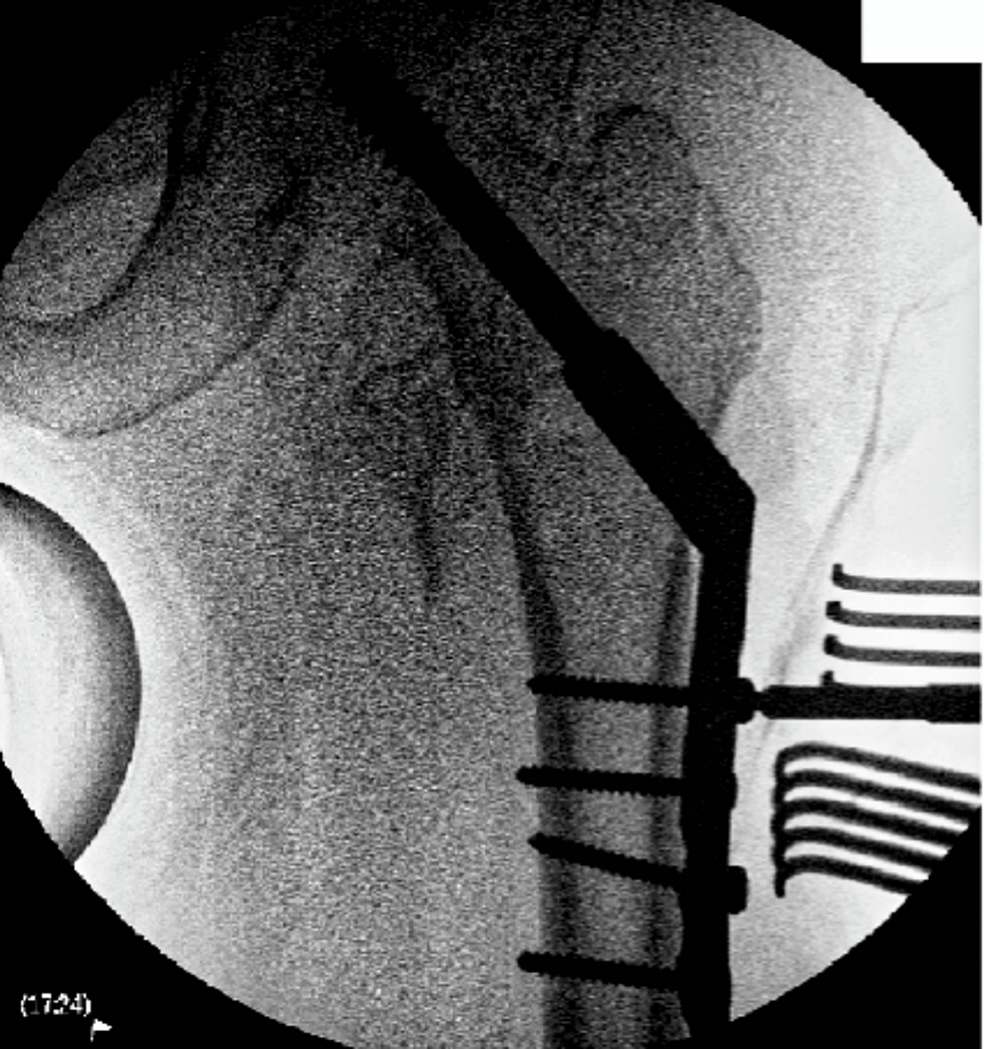
Intraoperative final X-ray of dynamic hip screw fixation.

A 76-year-female was referred to the on-call team after she had a fall and sustained an injury to her left lower limb. She was healthy and was independently mobile at home. She was on a direct oral anticoagulant (edoxaban) for her atrial fibrillation. Preliminary radiographs of her left hip showed an extracapsular proximal femur fracture. She was admitted and planned for a DHS fixation. Preoperative blood reports were all normal with haemoglobin of 124 g/L. She underwent fracture fixation 24 hours after her last dose of edoxaban which was stopped on admission. A DHS fixation was done under a general anaesthetic on a traction table (Figure 1).

A 10 cm skin incision was made distal to the flare of the greater trochanter along the long axis of the femoral shaft. Fascia lata was incised and vastus lateralis was split to reach the bone. The bone was cleared off any muscle using a periosteal elevator. A four-hole DHS was inserted after lag screw insertion and fixed with four cortical screws. Haemostasis was achieved throughout the procedure and the wound was thoroughly washed before closure. There were no active bleeders during the closure of the wound. The patient was stable throughout the procedure, and the surgery was uneventful.

**Figure 2:**
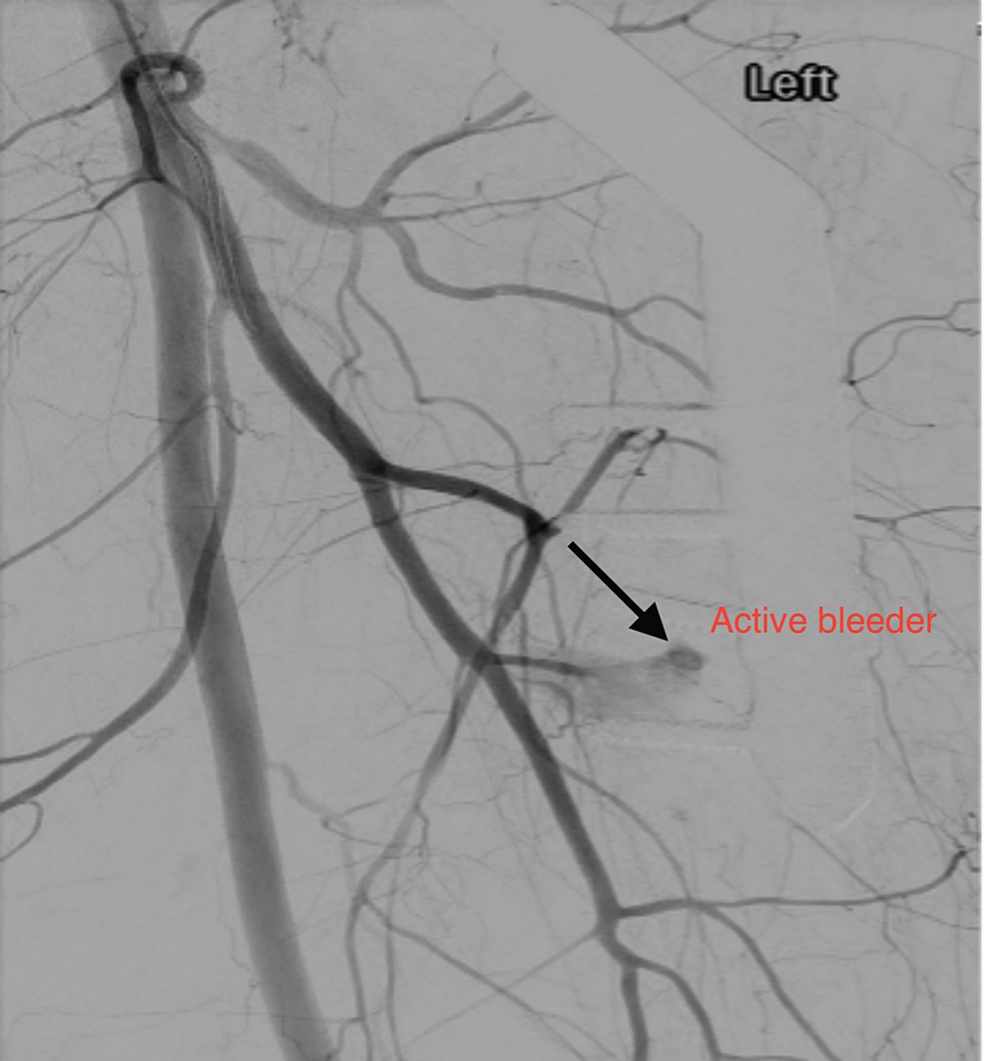
Active bleeding from one of the branches of the profunda femoris artery on CT angiogram of the left lower limb.

On the first postoperative day, she was well in herself, and the dressing over the surgical incision site was clean and dry. Her postoperative haemoglobin was 64 g/L. She was transfused two units of blood the same day. This brought her haemoglobin back up to 80 g/L. We also noticed bruising and swelling over the medial side of her thigh. The patient was clinically stable with normal vitals. CT angiography of her left lower limb was done which pointed out significant haemorrhage from a branch of the profunda femoris, which was in close proximity to the third screw tip (Figure 2).

**Figure 3:**
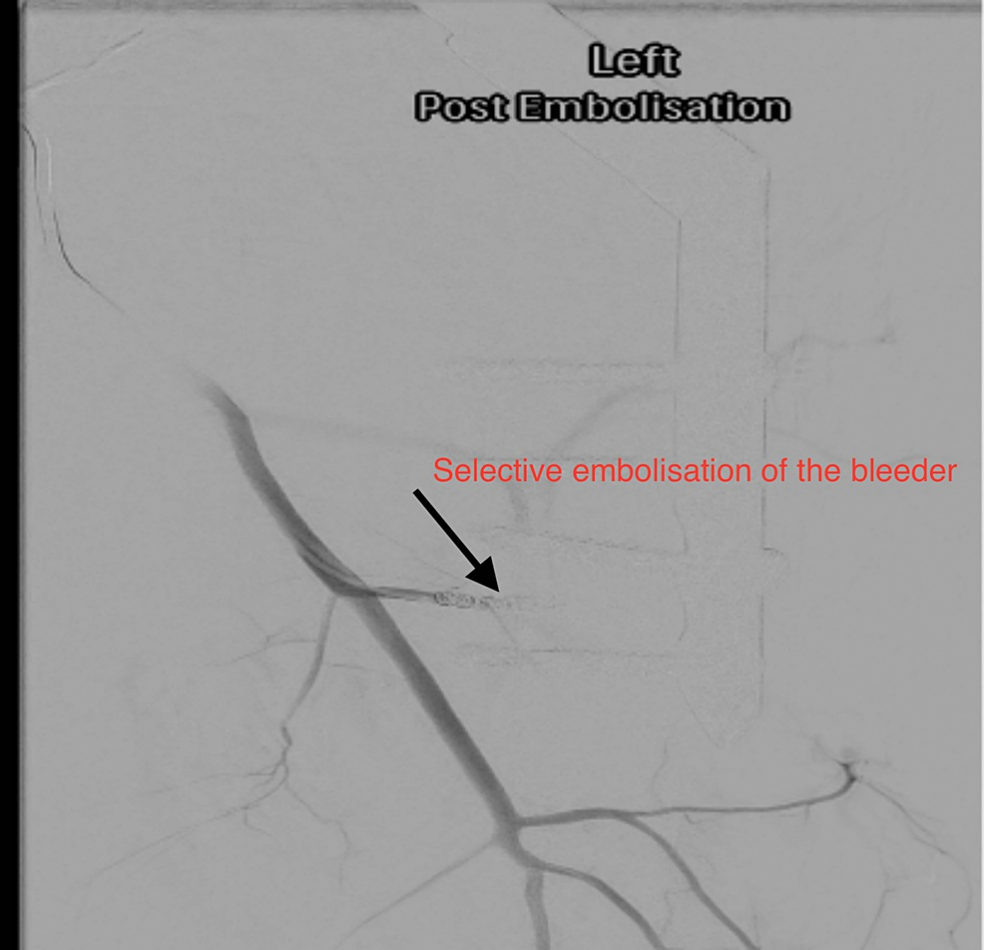
Post-embolisation images on CT angiogram.

After identifying the active bleeder, the patient underwent selective endovascular coil embolisation (Figure 3).

Post-embolisation, the patient was haemodynamically stable. The patient remained haemodynamically stable and was discharged once she cleared the stair assessment by a physiotherapist. Post-procedure haemoglobin level was 87 g/L, which was determined the next day.

## Discussion

With the increasing incidence of proximal femoral fractures, the need to pay attention to the treatment of complications has also increased [7]. Iatrogenic vascular injuries are more severe complications and are difficult to diagnose early [8]. Understanding the anatomical relationship between the femur and femoral artery along with its branches is critical in preventing iatrogenic vascular injuries. Sun et al. showed that the femoral artery along the medial side of the femur, especially PFA and deep femoral artery, were at a higher risk of injury as they lay within 13 mm of bone [9]. The femoral artery on the medial side of the femur was at risk to screw insertion or overshooting with the drill. Laohapoonrungsee et al. reported that the shorter drill bit and accurate screw length are vital in preventing injury to the PFA [10].

Pseudoaneurysms or haemorrhage of the PFA following orthopaedic bone fixation remain rare and are underreported [11]. The various mechanisms causing this include sharp bone fragments, protruding tip of cortical screws, and distal locking screws in the Gamma nail [10]. The acute vascular injury presents with tachycardia, hypotension, declining haematocrit, sudden swelling, and bruising over the thigh with pulsatile swelling [11]. This clinical picture presents when there has been a vascular insult by an overshooting drill or by the tip of a cortical screw [12].

Early diagnosis is critical in the management of iatrogenic vascular injuries. Imaging modalities that can help rule out vascular bleed are duplex ultrasonography, CT, CT contrast angiography, conventional angiography, and MRI. All these modalities help in localising the bleeding vessel. Interventional radiologists play a critical role in this multidisciplinary team approach. Embolization of the bleeding vessel is the treatment of choice. Giurazza et al. in a retrospective study of 14 patients who underwent endovascular embolisation for iatrogenic vascular injury following orthopaedic surgery concluded that endovascular embolization is both a safe and effective procedure in treating this subset of patients [13] and reducing the morbidity associated with surgical vascular repair [4]. Other treatment options include percutaneous ultrasound-guided thrombin injection into the lesion avoiding the need for transarterial catheterization or exposure of the PFA [14].

## Conclusions

Iatrogenic vascular injury can be difficult to diagnose and requires a high index of suspicion. Advanced imaging modalities are the surgeons’ best guide to locating the cause as well as treating them. Pseudoaneurysm or active haemorrhage should be suspected if a patient continues to have clinical signs and symptoms even after fluid resuscitation or blood transfusion. We would like to emphasise the careful use of drill bits and depth gauge. We recommend careful drilling of the second cortex as overpenetration by drill bit is a cause of iatrogenic vascular injury.
